# Supplementary material for: Time-to-Treatment of Oral Cancer and Potentially Malignant Oral Disorders: Findings in Malaysian Public Healthcare
Source: Dent J (Basel). 2022 Oct 24;10(11):199. doi: 10.3390/dj10110199 (PMC9689072; doi:10.3390/dj10110199)
Supplement: Supplementary file 1 [file dentistry-10-00199-s001.zip › Supplemental Table S1.pdf]

Table S1. Differences in the patient interval (in days) based on sociodemographic and clinical factors.

Characteristic		n	Interval Median (IQR) (In Days)	p-Value [1]
**Age**	<60	49	63 (21–181)	0.975
	>60	51	61 (21–180)	
**Gender**	Male	39	60 (19–102)	0.157
	Female	61	90 (27–181)	
**Race**	Malay	21	66 (30–150)	0.690
	Chinese	19	60 (21–360)	
	Indian	50	76 (23–180)	
	Indigenous	10	42 (9–92)	
**Location**	Urban	46	60 (19–181)	0.566
	Rural	54	90 (21–180)	
**Education**	None/Primary	45	63 (30–181)	0.224
	Secondary/Tertiary	55	61 (18–120)	
**Occupation**	Not employed	43	90 (16–183)	0.409
	Employed/Retired	57	61 (27–102)	
**Household income**	≤MYR 4360	90	63 (21–181)	0.428
	>MYR 4360	10	57 (21–90)	
**Anatomic site**	Buccal mucosa	54	70 (19–181)	0.182
	Tongue	25	66 (33–120)	
	Others [2]	21	31 (15–92)	
**Diagnosis**	OPMD	50	72 (19–120)	0.820
	Oral Cancer	47	61 (21–183)	

[1] Significant difference in patient interval between OPMD and oral cancer based on the Kruskal-Wallis H test with significance set to $p < 0.05$. [2] Consists of the alveolar, gingiva, lip, floor of mouth, palate, mandible, and other sites.
