# Supplementary material for: Time-to-Treatment of Oral Cancer and Potentially Malignant Oral Disorders: Findings in Malaysian Public Healthcare
Source: Dent J (Basel). 2022 Oct 24;10(11):199. doi: 10.3390/dj10110199 (PMC9689072; doi:10.3390/dj10110199)
Supplement: Supplementary file 1 [file dentistry-10-00199-s001.zip › Supplemental Table S2.pdf]

Table S2. Awareness and health-seeking behaviors of respondents.

Factors	%	Interval Median (IQR) (In Days)	*p*-Value
Ever heard about oral cancer before diagnosis?			
Yes	59.6	60 (15–180)	0.861 [1]
No	40.4	60 (23–150)	
Are dentists trained for oral cancer screening?			
Yes	53.5	60 (15–120)	0.595 [1]
No	46.5	60 (15–180)	
If attended annual dental check-ups before diagnosis?			
Yes	16.5	51 (23–90)	0.453[1]
No	83.5	60 (15–180)	
Type of first primary healthcare professional seen			
Dentist	43.9	0 (0–7)	0.730 [2]
Medical officer	56.1	0 (0–8)	

[1] Kruskal-Wallis H test to detect differences in T_1, with significance set to $p<0.05$. [2] Kruskal-Wallis H test to detect differences in T_2, with significance set to $p < 0.05$.
